# Prevalence and mortality of cancer among HIV-infected inpatients in Beijing, China

**DOI:** 10.1186/s12879-016-1416-3

**Published:** 2016-02-16

**Authors:** Jun Yang, Shu Su, Hongxin Zhao, Dennis Wang, Jiali Wang, Fujie Zhang, Yan Zhao

**Affiliations:** Cancer Hospital, Chinese Academy of Medical Sciences, Beijing, China; National Center for AIDS/STD Control and Prevention, Chinese Center for Disease Control and Prevention, Beijing, China; Beijing Ditan Hospital, Capital Medical University, Beijing, China; School of Public Health, Yale University, New Haven, CT USA

**Keywords:** HIV, Cancer, Concurrence, Prevalence, Mortality, Antiretroviral therapy, China

## Abstract

**Background:**

Cancer is responsible for elevated HIV-related morbidity and mortality. Research on HIV-infected patients with concurrent cancer is rare in China. The purpose of our study was to investigate the prevalence and risk factors associated with cancer among HIV-infected inpatients in Beijing, and to investigate the mortality and risk factors among HIV-infected inpatients with cancer.

**Methods:**

Hospital records from a total of 1946 HIV-infected patients were collected from the Beijing Ditan Hospital. The data, from 2008 to 2013, were collected retrospectively. The cancer diagnoses included AIDS-defining cancers (ADC) and non-AIDS defining cancers (NADC). Logistic regression was used to identify risk factors predicting the concurrence of cancer with HIV. Mortality was examined using Kaplan-Meier estimates and Cox proportional hazards models.

**Results:**

7.7 % (149 cases) of all HIV-infected inpatients had concurrent cancer at their first hospital admission; of those, 33.6 % (50 cases) had ADCs, and 66.4 % (99 cases) had NADCs. The most prevalent NADCs were Hodgkin’s lymphoma, gastrointestinal cancer, liver cancer, and lung cancer. Patients who did not accept antiretroviral therapy (ART) were more likely to suffer from cancer [AOR = 2.07 (1.42–3.01), *p* = 0.001]. Kaplan-Meier curves indicated that the survival probability of HIV-positive cancer patients was significantly lower than that of HIV-positive cancer-free patients (log-rank test, *p* < 0.001). For patients diagnosed with cancer, the mortality was also higher among those who did not receive ART [AHR = 2.19 (1.84–2.61), *p* < 0.001].

**Conclusion:**

The prevalence of cancer concurrence among hospitalized HIV-infected patients was 7.7 %. Concurrent cancer also increased mortality among HIV-infected patients. ART was protective against concurrent cancer as well as mortality among HIV-infected cancer patients. These results highlight the importance of promoting cancer screening and early ART initiation among HIV-infected patients.

## Background

People living with HIV/AIDS (PLWHA) are at higher risk of developing concurrent cancer due to the impairment of the immune system [1]. Cancer is in turn responsible for increased HIV-related morbidity and mortality [2, 3]. Cancers can be categorized by their associations with HIV/AIDS as AIDS-defining cancers (ADCs) and non-AIDS-defining cancers (NADCs) [4]. Generally, risk factors for developing cancer among PLWHA include low CD4 cell count or late stage of AIDS, co-infection with other viruses, cigarette smoking, alcohol consumption, and advanced age [5].

Globally, the wide application of antiretroviral therapy (ART), which has changed HIV/AIDS from a fatal disease to a chronic one, has contributed to a shift in cancer patterns among HIV-infected patients [6]. Evidence reveals that risk of ADCs has been dropping among HIV/AIDS patients since 1996 [6]. However, NADCs burden among HIV/AIDS patients are still on the rise [5–7] for reasons that remain unclear. In addition, although ART has been reported to be effective in lowering the risk of opportunistic infection and treating cancer-related diseases [8–10], evidence for the efficacy of ART in preventing the development of concurrent cancer among HIV-infected patients is lacking.

The mortality rate of ADCs has decreased while the mortality rate of NADCs has increased in the ART era [7, 11, 12]. While the former was always attributed to the wider application of ART [13, 14], deaths due to NADCs have been on the rise. Some studies attributed the increasing mortality of NADCs to lifestyle factors such as alcohol consumption and drug abuse [15], while others considered older age and lower CD4 counts to be the responsible factors [16].

In 2004, China started its free national ART program and began to provide free ART medications as well as related services to HIV-infected patients. The mortality of Chinese AIDS patients subsequently experienced a significant decline in the following decade [17]. However, very limited information is available on concurrent cancer among HIV/AIDS patients in China. Some HIV-infected patients were not identified until their CD4 counts decreased to less than 200 cells/mm^3^, after the onset of AIDS [17–19]. Late identification of HIV infection and under-resourced healthcare settings, especially in rural China, made concurrent cancer identification difficult. As a result, the prevalence of cancer concurrence among PLWHA was underestimated.

This study, based on a 5-year retrospective cohort at Beijing Ditan Hospital, focuses on the epidemiology of cancer among PLWHA. Our hypothesis was that ART was protective against concurrent cancer as well as cancer-related mortality among HIV-infected patients. We aimed to identify the risk factors associated with concurrent cancer among PLWHA and risk factors associated with mortality among PLWHA with cancer.

## Methods

### Study population

Beijing Ditan Hospital is one of largest specialized hospitals providing healthcare services to HIV-infected patients in China. As Beijing Ditan Hospital is a comprehensive, well-staffed, and well-equipped hospital with a professional reputation, HIV-infected patients from across China travel to the hospital to seek medical services. Therefore, the sample from Beijing Ditan Hospital is composed of a mix of HIV patients from all over the country.

### Data collection

Information on each patient was collected from his/her hospital records. Permission was obtained from Beijing Ditan Hospital for accessing the patient medical records. The follow-up data within the observational period were extracted from the national AIDS information system, which compiles compulsory reports of HIV cases as well as follow-up data every 6 months. Information collected in this system included demography, updated health status, CD4 counts, ART status, and survival status. The national AIDS information system is not publicly available. For the study, we applied the permission of data use for this study from National Center for AIDS/STD Control and Prevention, which took responsibility of managing the information system.

### Sampling criteria

**Fig. 1:**
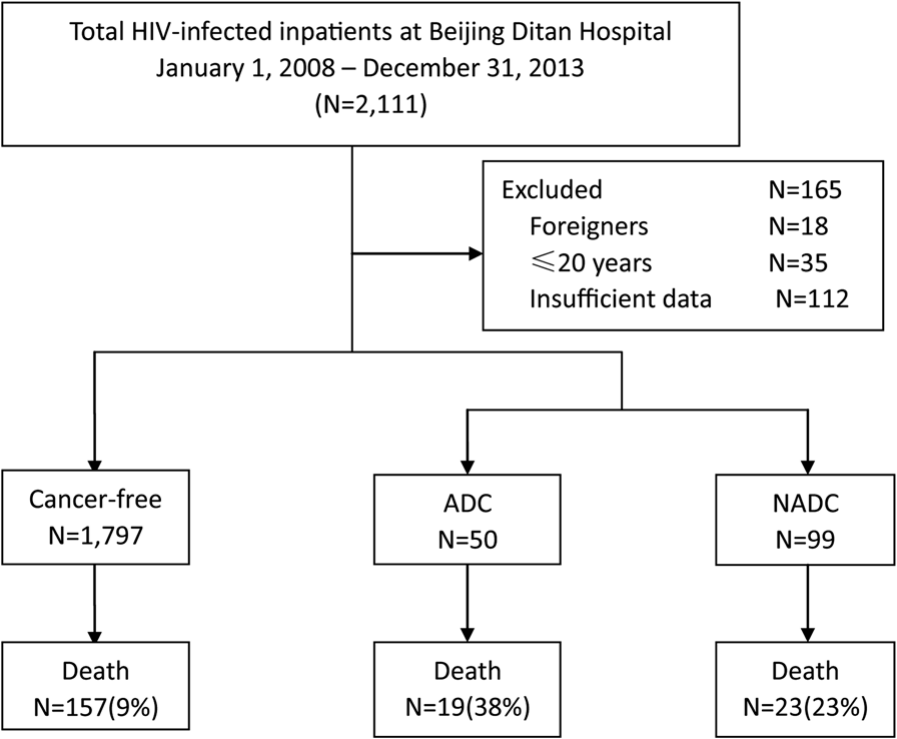
Framework of the study

We included all HIV-infected inpatients older than 20 who were admitted to Beijing Ditan Hospital between January 1, 2008 and December 31, 2013. Patients were considered to be enrolled in the study from the time of their first admission to Beijing Ditan Hospital until death or the end of the study period on December 31, 2013. Outpatient records were not included as the records did not include all of the necessary information. The retrospective data collection was performed from May 1 to June 30, 2014. The sampling framework for this study is shown in Fig. 1. Data from the national AIDS information system was linked to hospital data to determine their time of HIV diagnosis, first CD4 count after HIV diagnosis, subsequent CD4 record, the time of ART initiation and the death time.

### Definition of the variables

HIV infection was defined as being laboratory-confirmed using Western blot. As is widely accepted [20, 21], cancers were categorized as ADCs (Kaposi’s sarcoma, KS; non-Hodgkin’s lymphoma, NHL; and cervical cancer) or NADCs (cancers of the lung, liver, kidney, anus, head and neck, skin, Hodgkin’s lymphoma, etc.). Cancer was diagnosed by image examination and biomarker analysis and further verified by biopsy. The time of first cancer diagnosis was not collected in this study.

### Statistical analysis

Patients with no record of death were censored at the end of the study period on December 31, 2013. Continuous variables were summarized using median and interquartile range (IQR), and categorical variables were summarized using frequencies and percentages. Cross-tabulations with *χ*^2^ or Fisher’s exact tests were performed to relate variables of interest. A multivariate logistic regression model was used to identify factors of concurrent cancer at the first hospitalization. Kaplan-Meier curves were used to estimate the survival function during the study period. A multivariate Cox proportional hazards model was used to identify the prognostic risk of death among patients with cancer. The start time of Cox analysis was first HIV diagnosis. Updated CD4 counts and ART status were modelled as time-dependent covariates. CD4 count was kept as continuous variable. A *p*-value of less than 0.05 was considered significant in the final reduced multivariate model. Patients with missing data were kept in the table and classified in the ‘unknown’ group. Data was managed and analysed using the SAS 9.2 statistical software.

### Ethical considerations

This study was reviewed and approved by the Institutional Review Board of the National Center for AIDS/STD Control and Prevention of the Chinese Center for Disease Control and Prevention. All participants signed the routine informed consent form when they were admitted to the hospital and no further informed consent was required. Patient information was de-identified for data analysis.

## Results

From January 1, 2008 to December 31, 2013, a total of 2111 hospitalized HIV-infected patients’ records were incorporated into the study from their admissions to the hospital. 165 cases (18 foreigners, 35 below the age requirement, 112 without sufficient information) were excluded based on our inclusion criteria. Ultimately, 1946 patients were enrolled in this study; their records were analysed with a total of 59,016 patient-months of follow-up.

### Characteristics of the patients

**Fig. 2:**
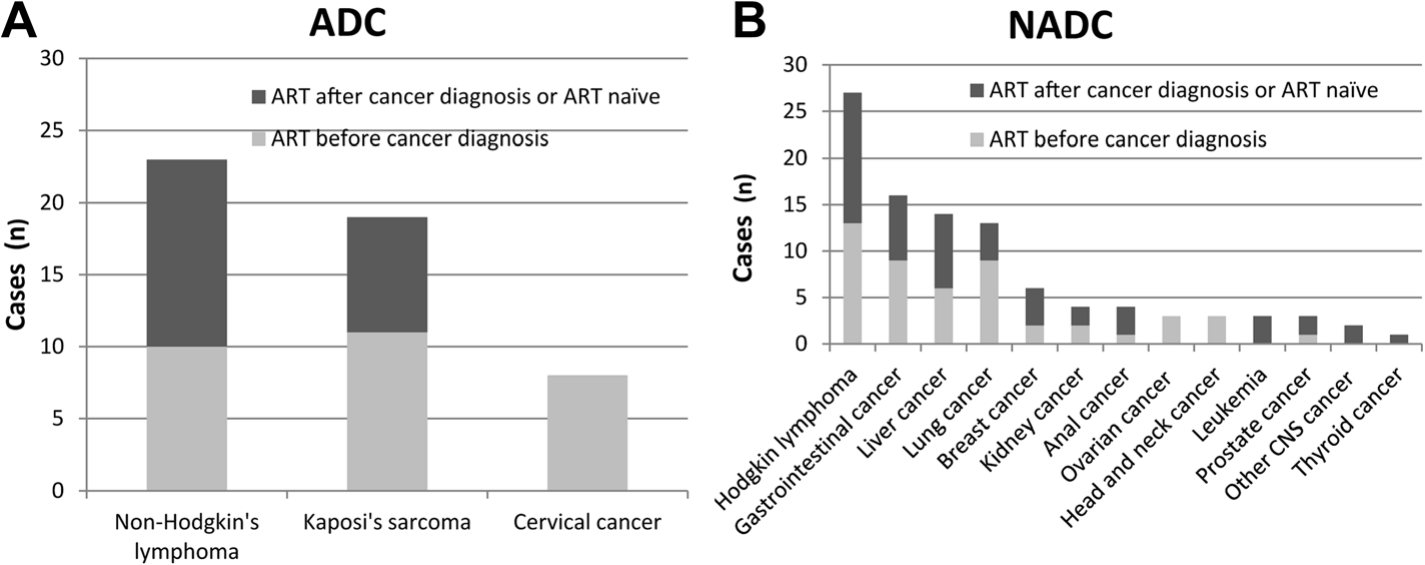
Distribution of cancers among HIV-infected inpatients with ADCs (A) and NADCs (B) at Beijing Ditan Hospital, January 1, 2008 – December 31, 2013

The baseline characteristics of ADC-concurrent, NADC-concurrent, and cancer-free HIV-infected inpatients are summarized in Table 1. 149 out of 1946 HIV-infected inpatients were diagnosed with concurrent cancer, accounting for 7.7 % of the study population. Among them, 33.6 % (50 patients) were diagnosed with ADCs, and 66.4 % (99 patients) were diagnosed with NADCs. The distribution of ADCs, as shown in Fig. 2, was 46 % NHL, 38 % KS, and 16 % cervical cancer. The most prevalent NADCs were Hodgkin’s lymphoma (27 %), gastrointestinal cancer (16 %), liver cancer (14 %), and lung cancer (13 %).

**Table 1:** Baseline clinical characteristics classified by cancer status among HIV-infected inpatients at Beijing Ditan Hospital, January 1, 2008 – December 31, 2013

Characteristics	Cancer-Free	Cancer			*p*-value*
	N (%)	N (%)			
	All	All	ADCs	NADCs	
Total	1797	149	50	99	
Gender					0.045
Female	359 (20.0)	40 (26.8)	15 (30.0)	25 (25.3)	
Male	1438 (80.0)	109 (73.2)	35 (70.0)	74 (74.7)	
Age (years)					
Median (IQR)	37 (30–45)	44 (37–53)	50 (39–57)	42 (36–49)	0.026
Age group					0.002
21–30	466 (25.9)	14 (9.4)	1 (2.0)	13 (15.4)	
31–40	621 (34.6)	48 (32.2)	14 (28.0)	34 (34.3)	
41–50	455 (25.3)	37 (24.8)	8 (16.0)	29 (29.3)	
51–60	171 (9.5)	32 (21.5)	16 (32.0)	16 (16.2)	
≥ 61	84 (4.7)	18 (12.1)	11 (22.0)	7 (7.1)	
HIV transmission mode					0.085
Men who have sex with men	635 (35.3)	35 (23.5)	6 (12.0)	29 (29.3)	
Injection drug use	81 (4.5)	2 (1.3)	0 (0.0)	2 (2.0)	
Heterosexual contact	800 (44.5)	83 (55.7)	31 (62.0)	52 (52.5)	
Blood transmission	224 (12.5)	24 (16.1)	12 (24.0)	12 (12.1)	
Unknown	57 (3.2)	5 (3.4)	1 (2.0)	4 (4.0)	
Median days from HIV diagnosis to the first hospital admission (IQR)	111 (27–663)	215 (49–760)	350 (58–847)	140 (36–402)	0.015
Median first CD4 cell count after HIV diagnosis (IQR) (cells/μl)	131 (36–265)	127 (51–369)	146 (85–402)	91 (20–255)	0.213
0–200	997 (55.5)	79 (53.0)	26 (56.0)	53 (51.5)	
201–350	422 (23.5)	29 (19.5)	10 (20.0)	19 (19.2)	
351–500	245 (13.6)	27 (18.1)	8 (16.0)	19 (19.2)	
> 500	133 (7.4)	14 (9.4)	6 (12.0)	8 (8.1)	
Median CD4 cell count at the first hospital admission (IQR) (cells/μl)	289 (135–441)	265 (115–374)	297 (201–396)	240 (95–351)	0.381
ART initiation before the first hospital admission	1206 (67.1)	78 (52.3)	29 (58.0)	49 (49.5)	0.004
The CD4 cell count at ART initiation	147 (60–273)	137 (66–265)	160 (90–297)	102 (36–228)	0.067

*p-value for comparing of cancer-free versus cancer group

There were nearly four times as many male patients (1547) as female patients (399), but 10 % of female HIV patients were diagnosed with cancer compared to 7 % of male HIV patients in the study (*p* < 0.05). The median age of HIV-infected patients without cancer was 37 years old, which was significantly younger than that of HIV-infected patients with cancer, at 44 years old (*p* < 0.001). 52.3 % of HIV-infected cancer patients initiated ART before admission at Beijing Ditan Hospital compared to 67.1 % of cancer-free patients (*p* = 0.004).

From the logistic regression model (Table 2), we identified ART status as a strong independent factor of cancer status among HIV-infected patients. Patients not on ART were more likely to have cancer than patients who were on ART [AOR = 2.07 (1.42–3.01), *p* = 0.001]. Advanced age is also associated with a slightly elevated risk of developing cancer [AOR = 1.05 (1.03–1.06), *p* = 0.001], while gender, route of HIV contraction, and CD4 cell count at HIV diagnosis were not found to be predictive of cancer status.

**Table 2:** Risk factors associated with concurrent cancer as calculated using logistic regression modeling among 1946 HIV-infected inpatients at Beijing Ditan Hospital, January 1, 2008 – December 31, 2013

Characteristic	OR (95 % CI)	*p*-value	Adjusted OR (95 % CI)	*p*-value
Total				
Gender				
Female	1.00		1.00	
Male	0.68 (0.47–0.99)	0.042	0.68 (0.45–1.05)	0.422
Age group every 10-year increase	1.05 (1.03–1.06)	0.011	1.05 (1.03–1.06)	0.001
HIV transmission mode				
Men who have sex with men	1.00		1.00	
Injection drug use	0.45 (0.11–1.90)	0.156	0.37 (0.09–1.61)	0.085
Heterosexual contact	1.88 (1.25–2.83)	0.037	1.42 (0.84–1.82)	0.524
Blood transmission	1.94 (1.13–3.34)	0.041	1.07 (0.86–1.32)	0.554
Unknown	1.59 (0.60–4.22)	0.263	0.89 (0.32–2.54)	0.734
First CD4 cell count after HIV diagnosis (cells/μl)				
0–200	1.00		1.00	
201–350	0.87 (0.56–1.35)	0.533	1.08 (0.67–1.75)	0.745
351–500	0.86 (0.55–1.35)	0.514	1.02 (0.63–1.64)	0.950
> 500	0.57 (0.32–1.02)	0.057	0.71 (0.39–1.30)	0.263
ART initiation before the first hospital admission				
Yes	1.00		1.00	
No	1.86 (1.33–2.60)	0.01	2.07 (1.42–3.01)	0.001

*OR* odds ratio, *CI* confidence interval

**Fig. 3:**
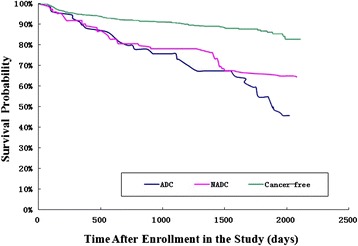
Kaplan-Meier survival curves for HIV-infected inpatients stratified by cancer type at Beijing Ditan Hospital, January 1, 2008 – December 31, 2013

Patients without cancer had significantly higher survival rates over the follow-up period than patients with ADCs or NADCs (log-rank test *p* < 0.001) (Fig. 3). A multivariate Cox proportional hazards analysis (Table 3) showed that patients with cancer who were not on ART were at higher risk for death [AHR = 2.19 (1.84–2.61), *p* < 0.001]. Time updating CD4 cell counts (from HIV diagnosis to the endpoint of this study) and advanced age did not affect mortality.

**Table 3:** Risk factors associated with mortality as calculated using Cox proportional hazards models among 149 inpatients with cancer at Beijing Ditan Hospital, January 1, 2008 – December 31, 2013

Characteristic	Number of Deaths (%)	Observed person-months	Mortality rate (deaths per 100 person-months)	HR(95 % CI)	*p*-value	Adjusted HR (95 % CI)	*p*-value
Total	42 (28.2)	5355	0.78	–	–	–	–
Gender							
Female	13 (32.5)	2012	0.65	1.00		1.00	
Male	29 (26.6)	3343	0.87	1.30 (0.67–2.53)	0.071	0.94 (0.41–2.13)	0.826
Age (every 10-year increase)	42 (28.2)	5355	0.78	1.02 (1.00–1.05)	0.923	1.02 (0.99–1.05)	0.912
HIV transmission mode							
Men who have sex with men	8 (22.9)	1029	0.78	1.00		1.00	
Injection drug use	0 (0.0)	110	0.00	–	–	–	–
Heterosexual contact	23 (27.7)	2468	0.93	1.17 (0.52–2.63)	0.227	1.27 (0.44–3.66)	0.363
Blood transmission	9 (37.5)	1601	0.56	0.58 (0.21–1.62)	0.189	0.78 (0.23–2.67)	0.314
Unknown	2 (40.0)	146	1.37	1.85 (0.39–8.76)	0.456	6.71 (0.67–66.89)	0.532
CD4 cell count^a^ (per cell/μl increase)	42 (28.2)	5355	0.78	0.99 (0.99–0.99)	<0.001	1.01 (0.99–1.02)	0.343
No ART initiation^a^	42 (28.2)	5355	0.78	2.52 (2.22–2.86)	<0.001	2.19 (1.84–2.61)	<0.001
Classification of cancer							
NADC	23 (23.2)	3151	0.73	1.00		1.00	
ADC	19 (38.0)	2204	0.86	1.12 (0.61–2.08)	0.121	0.86 (0.38–1.93)	0.082

*HR* hazard ratio, *CI* confidence interval

^a^Variables were modelled as time-dependent covariates

## Discussion

Research on HIV-infected patients with concurrent cancer is lacking in China [22]. The purpose of this study was to investigate the prevalence of cancer in HIV-infected inpatients and to identify the risk factors associated with cancer among PLWHA and with mortality among PLWHA with cancer.

Related factors for developing cancer among the HIV-infected population include low CD4 cell count, route of HIV contraction, co-infection with other viruses, late stage of AIDS, cigarette smoking, alcohol consumption, and advanced age [5]. The finding of advanced age as an independent predictor of developing cancer is consistent with previous studies [7]. In our study, patients with cancer were on average 7 years older than those without cancer (44 years versus 37 years, *p* = 0.026), suggesting that it may be meaningful to advocate regular cancer screening and care among older HIV patients in China.

The capacity for cancer screening and diagnosis largely depends on techniques such as biomarker analysis, CT and MRI scanning, endoscopy, and biopsy [23]. These methods are available at major hospitals, but many HIV patients in rural China may miss the opportunity to be screened for cancer due to the high cost burden or the inaccessibility of medical facilities or service providers.

The prevalence, of cancer among patients enrolled in our study was 7.7 % (149/1946). Data from the national registry indicated that the crude incidence rate of cancer in 2011 was 235/100,000 and that cancers of the lung, female breast, gastrointestinal tract, liver, and cervix were the most common malignancies in China [24]. In our HIV-positive population, the most prevalent NADCs were Hodgkin’s lymphoma, gastrointestinal cancer, liver cancer, and lung cancer, different from the national pattern.

Previous research in developed countries showed the decrease of the incidences of ADCs and NADCs in the post-ART era [25]. As China’s national ART program began later than those of most developed countries and some HIV-infected patients were first identified at a late stage with low CD4 counts [19], it is reasonable that China still experiences HIV-related cancer epidemics in the ART era [26]. It is therefore important to initiate surveillance to track changes in the incidences of ADCs and NADCs.

Effective ART was found to reduce some of the excess risk of developing cancer among HIV patients [27]. The ART rate among HIV patients diagnosed with cancer was lower than among cancer-free patients (52.3 % versus 67.1 %, *p* = 0.004). Patients without ART were more likely to get cancer than patients who accepted ART [2.07 (1.42–3.01), *p* = 0.001]. This result indicated that treatment could be beneficial in reducing the risk of cancer among HIV-infected patients. Additionally, reasons why patients who were not on ART even they were in the serious progression should be considered. Being very sick and near death reduced the likelihood that ART was prescribed by their physician.

Mortality rates in both the cancer-concurrent group as well as the cancer-free group were relatively high, in part because our study population was drawn from hospital admissions who were more likely to have advanced disease progression. Our finding that HIV-infected patients with cancer who received ART were less likely to experience premature death is encouraging. A large-scale and long-term cohort study in the U.S. found that overall cancer-related mortality among PLWHA experienced a major drop (from 302 to 29 per 1000 patients) between 1980 and 2006 [28]. Another U.S. cohort study reported that mortality rates among HIV patients with cancer who received ART were much lower than among HIV patients who did not, while the time of ART initiation, before or after cancer diagnosis, did not make a significant difference [3]. The Johns Hopkins AIDS Clinic cohort discovered no difference in mortality rates in ADCs and NADCs among patients who received ART [29].

### Limitations

Our study has a few limitations. The sample from Beijing Ditan Hospital could be biased because it is a referral center for infection disease treatment. HIV-infected patients admitted by Beijing Ditan Hospital came from all over China, but the study population cannot be considered a random sample as only those who had relatively severe complications and who could afford the medical expenses would come to this hospital. As a result, selection bias may have occurred, leading to the overestimation of the prevalence of concurrent cancer among HIV-infected patients.

There was another major weakness of this study. The time of first cancer diagnosis was not collected in this study. Patients were assessed concurrent cancer when they were firstly hospitalized in our study facility, Beijing Ditan hospital. We could not accurately describe the occurrence of cancer.

Due to the nature of the retrospective study, it was not possible for us to collect data on as many clinical variables as may have been needed for the study, such as viral load. Therefore, there exists the possibility of residual confounding due to data insufficiency. For example, the regression models did not include covariates such as alcohol dependence and co-infection with other viruses as in other studies, as they were not available in all of our routine clinical data.

## Conclusion

The concurrence of cancers among HIV-infected patients was 7.7 % in inpatients in our study hospital. Cancers increased mortality rates among HIV-infected patients, but ART was effective both in reducing the risk of cancer development among HIV-infected patients and in reducing the risk of cancer-related mortality. It is therefore important to increase cancer screening and to promote early initiation of ART among HIV-infected patients.
